# Droplet Digital PCR (ddPCR) Does Not Enhance the Sensitivity of Detection of Cytomegalovirus (CMV) DNA in Newborn Dried Blood Spots Evaluated in the Context of Newborn Congenital CMV (cCMV) Screening

**DOI:** 10.3390/ijns10010001

**Published:** 2023-12-20

**Authors:** Nelmary Hernandez-Alvarado, Craig J. Bierle, Mark R. Schleiss

**Affiliations:** Department of Pediatrics, Division of Pediatric Infectious Diseases, University of Minnesota Medical School, Minneapolis, MN 55455, USA; hernande@umn.edu (N.H.-A.); cjbierle@umn.edu (C.J.B.)

**Keywords:** congenital cytomegalovirus, digital droplet polymerase chain reaction, dried blood spots, newborn screening

## Abstract

Congenital cytomegalovirus (cCMV) infection is a leading cause of sensorineural hearing loss (SNHL) and neurodevelopmental disabilities in children worldwide. Some regions in the United States and Canada have implemented universal newborn screening for cCMV, which requires molecular diagnostic technologies for identifying cCMV, such as PCR testing of newborn dried blood spots (DBS). This study aimed to evaluate the sensitivity of droplet digital PCR (ddPCR) compared to quantitative real-time PCR to detect CMV DNA in newborn DBS. The limit of detection of various ddPCR primer/probe combinations (singleplex UL55-HEX, singleplex UL83-FAM, and multiplex UL55-HEX/UL83-FAM) was evaluated using the National Institute of Standards and Technology’s (NIST) CMV quantitative standard. Singleplex UL55-HEX ddPCR exhibited the lowest limit of detection among the primer/probe combinations tested for ddPCR. UL55 ddPCR was then compared to real-time PCR in 49 infants with confirmed cCMV identified through newborn screening for CMV in saliva swabs and confirmed by a urine test. The results showed that ddPCR was only positive for 59% (29 out of 49) of the cCMV infants, while real-time PCR was positive for 80% (39 out of 49). Due to its lower sensitivity and throughput, ddPCR may not be suitable for cCMV newborn screening.

## 1. Introduction

Congenital cytomegalovirus (cCMV) infection is a significant public health concern due to its potential to cause severe and permanent disabilities, including SNHL, intellectual disability, and vision impairment. CMV is the most common congenital viral infection, affecting an estimated 0.5 to 1% of all live births worldwide [1]. Despite its relatively high prevalence, cCMV often remains unrecognized and undiagnosed. Most infants born with cCMV do not show visible symptoms at birth but may develop lasting problems such as SNHL and developmental delays. Newborn screening has been proposed as a solution to enhance the detection of cCMV. Early identification allows for targeted follow-up and interventions based on the severity of the infection, which can significantly improve outcomes for affected children [2].

The newborn CMV screening process entails detecting CMV DNA in samples collected from infants soon after birth, necessitating sample collection before the baby reaches 21 days of age. A variety of sample types, including urine, saliva, and DBS, can be used for screening. Public health laboratories nationwide perform screening of metabolic abnormalities using DBS, making this specimen an inexpensive, accessible sample for newborn screening. However, the CMV viral load in blood is typically lower than that in saliva and urine, and the limited quantity of biological material present in a DBS is a technical barrier. Urine and saliva can be collected non-invasively and have high sensitivity and specificity for CMV detection [3]. Nonetheless, collecting these sample types is expensive compared to DBS, requiring additional healthcare provider time and infrastructure to deliver the samples to public health laboratories [4]. Public health prioritizes the best use of limited healthcare dollars and encourages using existing infrastructure in a newborn screening program when possible.

In July 2013, Utah became the first state to implement targeted CMV screening in the United States. For targeted CMV screening, a diagnostic test is performed whenever an infant refers on their newborn hearing screening test, with the goal of testing the infant before three weeks of age [5]. Other states have subsequently passed similar legislation, and some hospitals and audiologists across the USA have implemented this targeted CMV screening approach, but it is not the standard of care universally. In Ontario, as part of the Infant Hearing Program, universal screening for CMV has been offered for all babies born after 29 July 2019 [6]. Minnesota started a universal newborn CMV screening program in February 2023 [7]. Connecticut has recently announced a plan to adopt universal cCMV screening, and New York State has an ongoing study of universal cCMV screening. All states and provinces currently engaged in universal cCMV screening are using DBS to screen for CMV by PCR detection.

Given the technical challenges of using DBS for cCMV screening, a highly sensitive approach for molecular testing should be implemented. Droplet digital PCR (ddPCR) has been proposed for monitoring CMV in the blood of hematopoietic stem cell transplant patients [8]. This technique enables the absolute quantification of nucleic acids in clinical samples by partitioning a sample into thousands of tiny individual PCR reactions using water-oil emulsion droplets [9]. PCR products are isolated within individual droplets, generating a fluorescent signal when fluorescent DNA-binding dyes intercalate into the PCR product or fluorescent probes are hydrolyzed. The relative endpoint fluorescent signal determines whether the target sequence is present or absent in each droplet [10]. One of the key benefits of ddPCR is its higher precision because it allows absolute quantification of the target nucleic acid without the need for a standard curve.

It is hypothesized that ddPCR can have greater sensitivity than real-time quantitative PCR (qPCR) because it would detect low levels of target nucleic acids in complex samples due to a higher tolerance for inhibitors, insofar as each droplet contains a single or few copies of the target nucleic acid [9]. Partitioning the samples reduces the impact of target competition, making PCR amplification less susceptible to inhibition. This partitioning significantly enhances the discriminatory capability of assays that vary by a single nucleotide. Previous studies with infectious diseases like hepatitis D, HPV, and SARS-CoV-2 have found better sensitivity than qPCR, especially in crude lysates [11,12,13]. CMV studies comparing ddPCR with real-time PCR using standards and plasma samples found that ddPCR has less variability relative to real-time PCR for high viral loads but does not improve the sensitivity compared to real-time PCR [8,14,15]. In a recent study, digital PCR was utilized to establish a lower limit of detection. However, it involved a pre-amplification step, similar to nested PCR, consisting of 10–20 cycles [16]. It is important to note that such amplification steps carry a higher risk of contamination during the transfer of first-round products to the second tube for the subsequent round of amplification [17].

This study aimed to compare the performance of ddPCR and qPCR in detecting and quantifying CMV DNA in clinical samples, particularly in samples employed in the diagnosis of CMV at birth. We compared the sensitivity between ddPCR and a previously validated real-time PCR assay for amplifying CMV DNA eluted from DBS samples using two different sets of clinical samples. The first set consisted of samples obtained from infants suspected of having cCMV. In contrast, the second set of samples was collected as part of an ongoing universal screening study, including samples from infants who had confirmed cCMV.

## 2. Materials and Methods

### 2.1. Clinical Samples

This study utilized two cohorts of clinical samples. The first group consisted of DBS samples obtained from children who exhibited signs suggestive of congenital CMV infection but were beyond 21 days of age at the onset of clinical evaluation. These children were referred by clinicians for CMV DNA testing on their archived newborn DBS, following parental consent, in an attempt to retrospectively diagnose cCMV. The archived DBS were maintained at a temperature of −80 °C. This sample set was previously tested by real-time PCR using UL83 primers (Table 1). Twenty-nine DBS samples from children aged between 1 month and 6 years were analyzed after their parents or legal guardians requested access to the archived newborn DBS from their respective newborn screening (NBS) programs. Among the twenty-nine children, 10 (34%) were positive for CMV in their DBS, while 19 (66%) were negative for CMV as determined by real-time PCR.

**Figure 1:**
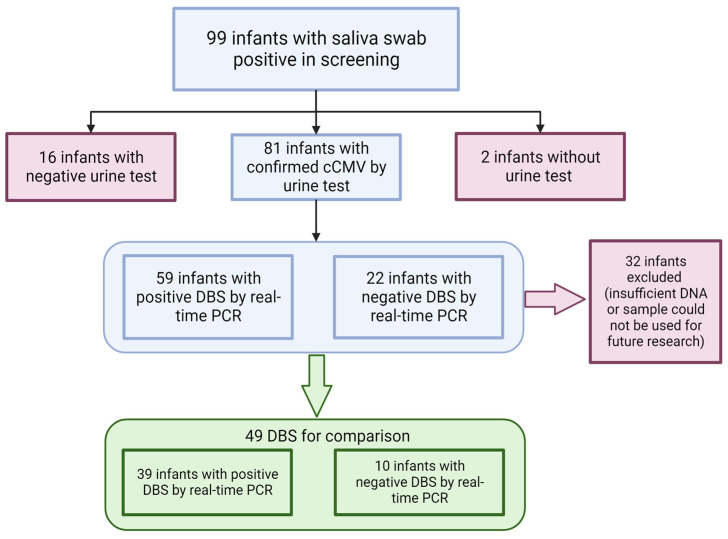
Flowchart of sample selection from the universal screening. The flow chart details the samples employed in the study obtained through universal screening. Red boxes signify samples excluded from this study, while green boxes indicate the samples included in the analysis.

The second set of samples was collected from infants enrolled in a clinical study titled “Diagnosing Congenital CMV Infection in Newborns as a Model for Universal Screening and Early Intervention” (IRB protocol number: 1507M76904DBS) [18]. As part of the study, saliva swabs and DBSs were collected and used to assay CMV DNA using real-time PCR with a UL83 (corresponding to CMV gene UL83, encoding the pp65 tegument phosphoprotein) primer set (Table 1). Informed consent was obtained between the child’s birth and the discharge from the hospital, ensuring that samples were collected no later than 21 days postpartum. Upon obtaining parental consent, saliva samples were collected from newborns, and DBSs were delivered from the Minnesota Department of Health to be tested at the University of Minnesota laboratory. Among the infants screened, 99 tested positive for CMV DNA in their saliva. Out of 99 saliva-positive infants, 81 (82%) were confirmed to have cCMV with a positive urine test during a clinical evaluation. Sixteen infants (16%) were classified as false positives for saliva because their follow-up confirmatory urine PCR test was negative (Figure 1). For two infants (2%), we were unable to confirm their cCMV status due to missing clinical information. DNA from the DBS was extracted within 2 weeks of collection. The eluted DNA underwent storage at 4 °C during qPCR testing and was later preserved at −80 °C until the completion of the ddPCR experiments. Out of the 81 infants that screened positive for saliva swab and were confirmed as having cCMV, 59 (73%) also tested positive for CMV DNA in the DBS screening using real-time PCR with UL83 primers, while 22 infants (27%) tested negative.

To evaluate the performance of ddPCR, we utilized residual DNA extracted from DBS samples from infants who tested positive for CMV in their saliva and urine samples. DBSs from 32 individuals were not included in this evaluation due to insufficient material for testing or objections from legal guardians to using the samples for future research. Of the remaining 49 infants included in this evaluation, 39 (80%) had positive DBS using real-time PCR with UL83 primers, whereas 10 (20%) were negative for DBS (Figure 1). We also utilized DBS DNA samples from 57 infants who were identified as CMV-negative during the universal screening study conducted using both saliva and DBS samples. These CMV-negative samples served as negative controls in our analysis.

### 2.2. DNA Extraction

DNA was extracted from three 3 mm punches using the QIAcube HT extractor with the QIAamp 96 DNA QIAcube kit (both by Qiagen) with slight modifications as previously described [18]. Briefly, 240 μL of ATL buffer (Qiagen, Hilden, Germany) with 10% proteinase K solution was added to each DBS sample and digested overnight in a Thermomixer incubator at 56 °C at 400 rpm. After samples were cooled to room temperature and spun at 700 RCF for 5 min, the digested material was transferred to a deep-well 96-well plate (S-block from Qiagen), and automated extraction was carried out with the QIAamp 96 DNA v1 protocol with one modification, specifically, to elute with 100 µL of molecular-grade water (Gibco, Billings, MT, USA).

### 2.3. Viral Load Determination by qPCR

PCR testing of DBS DNA was performed in triplicate, as previously described [19]. Multiplex qPCR was performed using the UL83 and NRAS primers shown in Table 1.

The standard curve for NRAS was generated using five 10-fold dilutions starting with 200,000 to 20 pg/µL of human genomic DNA (Roche, Basel, Switzerland). A DBS was considered positive for CMV if at least 2 of 3 replicates were positive with a crossing-point (Cp) of 40 or less. For specimens that were positive for 1 out of 3 replicates, a second PCR was performed. For samples tested twice, the specimen was considered positive if at least 2 out of 6 replicates were positive for CMV DNA.

### 2.4. Viral Load Determination by ddPCR

The ddPCR reaction was performed using ddPCR Multiplex Supermix (Bio-Rad, Hercules, CA, USA), using primers and probes as described in Table 1, with 10 units of Hind III and 10 µL of template. The final concentration of the primers was 900 nM and 250 nM for the UL55-Hex [8] and UL83-FAM reactions, respectively. After droplet generation with the QX200 Droplet Generator, the ddPCR was run using a C1000 Touch thermal cycler with the cycling conditions as follows: 95 °C for 10 min, 45 cycles of 94 °C for 30 s, 60 °C for 1 min, and 98 °C for 10 min. The results were read in the QX100 Droplet Reader and analyzed with the QuantaSoft Analysis Pro software (v1.0, Bio-Rad, Hercules, CA, USA).

## 3. Results

### 3.1. Limit of Detection of the ddPCR Assay

To assess the analytical sensitivity of UL83 and UL55 ddPCR, we conducted experiments using dilutions of HCMV DNA in separate ddPCR reactions. The DNA template utilized in this study was CMV TowneΔ147 BAC DNA obtained from the National Institute of Standards Technology (NIST) [20]. Each DNA aliquot across the concentration range was tested twenty times across ten different experiments, with two replicates for each experiment. The concentrations of TowneΔ147 BAC DNA ranged from 2245 to 0.3 copies per PCR reaction, employing 2-fold dilutions (Table 2). DNA dilution series were prepared freshly on the day of the assay. We determined the analytical limit of detection (LOD) with singleplex and multiplex reactions for UL83-FAM and UL55-HEX. A reaction was considered positive when a minimum of two droplets were above the threshold of detection of the endpoint fluorescence signal. We used the same standard to compare the real-time qPCR UL83 reaction with the ddPCR reactions.

Table 2 shows the number of times the dilution was positive and the average copies per PCR reaction calculated by the qPCR or ddPCR for each CMV TowneΔ147 BAC DNA concentration. The UL83 real-time PCR reaction was positive 19 out of 20 times, at 1.1 copies per PCR reaction. Singleplex UL55-HEX was positive in 100% (20 out of 20) of the replicates, with a concentration of 8.8 copies per PCR reaction. UL83-FAM was positive for 95% (19 out of 20 reactions tested) in the same concentration (8.8 copies per PCR reaction). The LOD was defined as the lowest concentration that resulted in at least 95% positive replicates [21]. Therefore, the limit of detection for UL55-HEX and UL83-FAM was 8.8 copies in the reaction and 1.1 copies per PCR reaction for real-time qPCR. In our test, UL55-HEX appeared to be slightly more effective in detecting DNA at a concentration of 8.8 copies per PCR reaction with 100% (20 out of 20) of the replicates. The LOD for the multiplex reaction was 17.5 CMV copies in the PCR reaction; both targets were positive 20 out of 20 times (100%) for a value of 17.5 CMV copies per reaction (Table 2).

**Figure 2:**
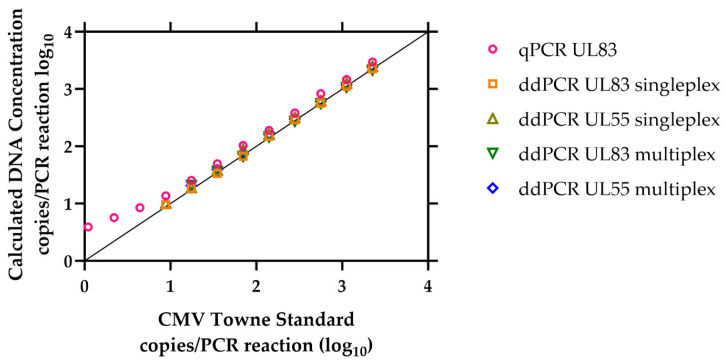
Quantification of CMV TowneΔ147 BAC DNA by real-time qPCR and ddPCR. The graph displays the average calculated DNA concentration for qPCR UL83, ddPCR singleplex, ddPCR UL55 singleplex, and multiplex UL83/UL55, using various dilutions from CMV TowneΔ147 BAC DNA. The data points included in the graph are only within the range of the limit of detection for each reaction. Additionally, a line of identity is depicted in black, representing points where x and y coordinates are equal.

Accuracy is the proximity of measurement results to the true value or an accepted reference value [22]. In our case, the reference values were the dilutions from CMV TowneΔ147 BAC DNA provided by NIST, as shown in the first column of Table 2. The certified values were derived as a consensus result from five PCR assays performed using a ddPCR system, along with a direct measurement of the mean droplet volume [20]. The calculated average copies per reaction (Figure 2) closely aligned with the reference values, indicating good accuracy. For all the ddPCR reactions, the calculated DNA concentrations were nearly identical to the reference values, with data points overlapping each other along the identity line (y = x), as expected for accurate measurements against a reference. However, for the real-time qPCR, there was a tendency to overestimate the DNA concentration by about 1.5-fold at concentrations of 8.8 copies per reaction or higher. Moreover, the accuracy decreased at lower concentrations (Figure 2), with the biggest difference of 3.6-fold noted at 1.1 copies per reaction.

### 3.2. Clinical Samples

We received consent to test DBS samples from 29 children suspected of having a cCMV infection. Our goal was to examine these samples for evidence of CMV DNA. Clinicians referred these children to us because they exhibited symptoms consistent with cCMV complications, such as SNHL, abnormal MRI results, vision loss, and/or developmental delays. We performed real-time PCR testing on the DBS samples using UL83 primers and a probe (Table 1). Out of the 29 samples, 10 (34.5%) tested positive for CMV DNA using real-time PCR (Table 3). A previous study on real-time PCR with the UL83 primers reported a sensitivity of 73.2% with a 95% confidence interval ranging from 60.4% to 83.0% [18].

To determine if any CMV DNA-positive cases were missed by real-time PCR, we conducted further testing using ddPCR. We compared the results of real-time PCR with ddPCR using singleplex assays for UL55 and UL83 and a multiplex assay for both UL55 and UL83. None of the DBS samples from children that had previously tested negative by real-time PCR showed positive results in any of the ddPCR assays (Table 3). Out of the ten samples that were positive by real-time PCR, six (samples #10, 14, 16, 18, 20, and 22) were positive for ddPCR in all three different reactions tested (Table 3). Among the remaining four samples, one (samples #11 and 29) tested negative for CMV DNA in all three ddPCR reactions. Additionally, sample #13 tested positive in both singleplex UL83 and UL55 but negative in the multiplex reaction. Sample #5 was positive for the UL55 channel in the multiplex reaction. Overall, among the ddPCR reactions that we tested, the UL55 primer set identified seven DBSs positive for CMV DNA across both multiplex and singleplex reactions. In comparison, the UL83 primer set identified six and seven positive DBSs in the multiplex and singleplex setups, respectively (Table 3).

Most of the viral loads previously calculated by real-time qPCR of DBS using UL83 primers were low. However, one sample (sample #10) showed a high viral load at 1630 copies in the reaction; this sample also tested positive in all ddPCR reactions. Interestingly, even though the lowest viral load estimated by real-time qPCR of DBS was only 1.4 copies per PCR reaction, it also tested positive in all ddPCR reactions. Overall, the DNA copy number calculations for the positive real-time qPCR and ddPCR samples were similar, except for sample 10, where the real-time qPCR calculated over 1000 copies in the reaction, while the ddPCR quantification for this sample ranged from 72–105.5 copies/reaction.

Next, we examined CMV ddPCR using DBS from infants with proven cCMV enrolled in a universal screening study (“Diagnosing Congenital CMV Infection in Newborns as a Model for Universal Screening and Early Intervention”; University of Minnesota IRB protocol number 1507M76904DBS). Unlike the previous set of clinical samples, we only had sufficient DNA remaining from our previous universal study for a single PCR comparison. We therefore decided to use singleplex UL55 ddPCR because, among the ddPCR reactions that we tested, UL55 had a lower limit of detection (LOD) and identified more children with cCMV in the previous set of clinical samples. We used the same DNA eluate that was used for the real-time PCR with UL83 for the UL55 ddPCR.

We included in our analysis 49 infants who had tested positive for CMV in their saliva during the screening program, had a confirmed diagnosis of cCMV based on a urine PCR test within the first three weeks of life, and had samples available for testing (Figure 1). Out of the 49 infants included in this analysis, 39 (80%) tested positive for CMV in their DBS samples using real-time polymerase chain reaction (PCR), while 10 (20%) tested negative. This particular set of samples offered a valuable opportunity to investigate whether ddPCR could enhance CMV detection in DNA eluted from DBS compared to real-time PCR, specifically in real-life samples from infants diagnosed with cCMV but who initially tested negative using real-time PCR.

Out of the 39 infants diagnosed with cCMV that were positive for DBS CMV DNA using real-time PCR, 29 infants also tested positive for CMV using ddPCR, while 10 infants tested negative. In addition, there were 10 infants who initially tested negative for CMV in their DBS samples using real-time PCR. Importantly, these infants tested positive during screening using saliva swabs, and the presence of cCMV was confirmed through a clinical standard-of-care urine PCR diagnostic test—they therefore clearly had cCMV infection. However, when these infants were tested with ddPCR, they still showed negative results for CMV in their DBS samples, as shown in Table 4. Thus, ddPCR failed to identify any infants with proven cCMV that had also screened negative using real-time DBS PCR (but were, as noted above, saliva-positive).

Overall, real-time PCR yielded positive results for 39 out of 49 DBS samples (80%) from infants with cCMV, while ddPCR showed positive results for 29 out of 49 DBS samples (59%) from the same cohort. Since both methods (real-time PCR and ddPCR) were performed on the same subjects, the McNemar test was used to evaluate the differences in diagnostic performance. The results revealed that real-time PCR yielded significantly more positive results (39/50 = 80%) than ddPCR (29/49 = 59%) in DBS samples (McNemar test, *p* = 0.0044).

## 4. Discussion

The diagnosis of cCMV at or shortly after birth is crucial for early intervention and improved outcomes for affected infants. In this study, the performance of ddPCR was compared to real-time PCR in detecting CMV DNA in clinical samples. First, the analytical sensitivity of UL83 and UL55 ddPCR singleplex and multiplex, as well as real-time qPCR using UL83 primers, was determined by testing different dilutions of NIST standard DNA. The LOD for real-time qPCR was determined to be 1.1 copies per PCR reaction, with 95% (19 out of 20) replicates demonstrating positive results. For ddPCR, the results showed that both targets had a LOD of 8.8 copies in the singleplex reaction, as defined by the lowest concentration that results in at least 95% positive replicates [21]. However, the results were not completely equal because UL55 was positive for 100% (20 out of 20); in comparison, UL83 was positive for 95% (19 out of 20) of the replicates with a concentration of 8.8 copies per PCR reaction (Table 2).

The multiplex reaction had a limit of detection of 17.5 CMV copies in the PCR reaction for both UL55 and UL83 targets. This was because both targets were positive in all 20 tests (100%) at a concentration of 17.5 CMV copies per reaction (Table 2). The calculated average number of copies per reaction was very similar to the reference values, indicating good accuracy, as shown in previous studies [8,14,15]. However, the calculated concentrations from real-time qPCR tend to be slightly higher than the reference material, and this trend was more pronounced when dealing with values of less than ten copies per reaction (Figure 1).

Next, we used DBS samples from two sets of clinical samples. The first group consisted of DBS from 29 children suspected of having cCMV. The reason these children underwent CMV DBS testing was that they had been referred by physicians with clinical presentations felt to be compatible with cCMV infection. Since these children were >21 days of age, viruria (DNAuria) could not reliably prove they had cCMV infection, necessitating examination, as available, of the DBS. Since we only had DBS samples available and lacked alternative samples for confirming cCMV, we compared our previous results obtained using real-time PCR with the results obtained from ddPCR reactions. We had enough DNA to compare the three ddPCR reactions: singleplex UL83, singleplex UL55, and multiplex UL83/UL55.

The results of these analyses were generally consistent with the LOD results. Among the ddPCR reactions we conducted, the UL55 primer set demonstrated a slightly better overall sensitivity for the detection of CMV DNA. Out of the twenty-nine samples tested, 10 (34.5%) were positive for CMV DNA using real-time PCR. Among these positive samples, seven tested positive for both singleplex and multiplex UL55 primers, as well as singleplex UL83. On the other hand, when using UL83 primers in the multiplex set-up, six samples showed positive results (Table 3). Furthermore, and notably, all three of the ddPCR assays yielded negative results for the nineteen samples collected from children who had previously tested negative using real-time PCR (Table 3).

The second group included DBS from 49 infants who had tested positive for CMV in their saliva during a universal screening study and had a confirmed diagnosis of cCMV based on a urine PCR test obtained within the first three weeks of life. This set of clinical samples presented a valuable opportunity to compare the performance of ddPCR and real-time PCR in confirmed cases of cCMV and, specifically, to examine whether ddPCR identified cases missed by real-time PCR, since some of these cases had been DBS screen-negative using the real-time assay.

Initially, using real-time PCR, 39 out of 49 (80%) infants diagnosed with cCMV tested positive for CMV in their DBS samples. Our goal was to determine if ddPCR could identify any confirmed cases of cCMV that had been missed by real-time PCR in the DBS samples. However, ddPCR did not detect any cCMV-infected infants that had tested as negative in the DBS samples using real-time PCR. Moreover, ddPCR only yielded positive results for 29 out of the 49 cCMV-infants, which accounted for 59% of the cases. In contrast, real-time PCR was positive for 39 out of 49 infants, representing 80% of the children (Table 4). The sensitivity of ddPCR in detecting cCMV in DBS samples was lower compared to real-time PCR (78% vs. 58%, assessed using the McNemar test, with *p* = 0.0044). To ensure the specificity of the ddPCR UL55 assay, we tested 57 DBS DNA samples from infants who had tested negative for CMV in both saliva and DBS during the screening study. In this validation, all 57 negative controls tested negative for the UL55 gene using ddPCR.

A previous study has suggested that the lower sensitivity of ddPCR compared to real-time PCR in detecting CMV DNA may be due to the lower input of template DNA [14]. ddPCR platforms have a lower volume limit than qPCR, which may result in lower template volume, and for any given sample, this limitation may lower assay sensitivity. In the current study, there was equal input of template DNA for our samples for both ddPCR and real-time PCR assays per well. However, for real-time PCR, we used technical replicates. We propose that this approach increases the total volume of DNA used for the assay and the likelihood of detecting viral templates if there are only a few copies in the DNA eluate. The ddPCR platform involves the preparation of the master mix reaction in one well, followed by droplet generation, transfer to another well, and subsequent droplet analysis via the flow of oil after amplification. It is important to note that the potential loss of reaction components or droplets during each of these steps may contribute to a decreased sensitivity when comparing both assays, even when the same DNA eluate and input volume per PCR reaction are utilized.

There has been speculation among researchers that ddPCR may offer advantages in processing “real-life” samples, despite an equal or higher LOD using laboratory standards, compared to other PCR platforms. Specifically, ddPCR has been suggested to be more resilient to mismatches in base pairs and to the PCR inhibitors that may be present in clinical samples [23], as compared to reference materials. However, our study did not observe such advantages in the context of the identification of CMV DNA in samples used for newborn screening. While there is considerable genetic variability in CMV sequence amongst clinical isolates [24], the primer and probe sequences used in clinical testing are generally designed to target conserved regions of the virus genome. Although ddPCR may be advantageous for detecting target sequences in rapidly mutating viruses such as HIV or SARS-CoV-2 [23], our findings suggest this is not necessarily the case for CMV.

We found interesting similarities and contrasts when comparing our study with other reports. One previous study focused on the sensitivity and reproducibility of CMV DNA between ddPCR and qPCR using whole blood samples [25]. They concluded that ddPCR and qPCR exhibited similar sensitivity. Additionally, they examined blood and urine specimens from neonates with cCMV and found no significant difference in CMV DNA loads measured by ddPCR and qPCR. In contrast to this study, our research specifically investigated the sensitivity of ddPCR in detecting CMV DNA in DBS samples collected at birth. Our findings revealed that ddPCR had lower sensitivity compared to real-time PCR for detecting CMV DNA in newborn DBS samples.

Another recently published paper concentrated on modified extraction methods for DBS samples using real-time PCR and ddPCR [26]. These invesigators tested these methods on various CMV-spiked samples and found a successful reduction of DBS starting material without compromising sensitivity. However, the modified methods demonstrated equivalent analytical sensitivity to the original method, consistently detecting CMV at high viral loads but inconsistently at low levels of viral DNA. Notably, they observed that the sensitivity of CMV detection using ddPCR did not improve at any viral concentration compared to real-time PCR. False-positive results were also observed with ddPCR. At the 2021 APHL conference, data on a ddPCR assay for CMV detection was presented [27], validated with spiked urine samples, demonstrating 95.7% concordance with qPCR. Generally concordant results were noted comparing qPCR and ddPCR in three DBS previously identified to be positive for CMV DNA in that study; further validation is awaited.

Typically, newborn screening assays aim for as close to 100% sensitivity as is feasible. False-positive results are considered acceptable in order to ensure that a *bona fide* case of disease is never missed. Although our studies with DBS-based real-time PCR for diagnosis of cCMV demonstrated substantially enhanced sensitivity [18] for identification of CMV DNA compared to previous reports [28], DBS testing still poses sensitivity challenges, which were observed in both real-time and ddPCR in the current study. This is probably due to a combination of factors, including a lower CMV viral load in blood than in saliva and/or urine and the limited amount of blood that can be recovered from three 3 mm punches from the DBS card. While saliva and urine are more sensitive, collecting these specimens in the newborn nursery is cumbersome and costly, particularly if applied to universal screening. For some screening studies, successful reports have highlighted the implementation of screening with urine collected on dried filter papers as a cost-effective and convenient approach to cCMV testing, and this approach is predicted to have a higher sensitivity for universal screening [29] than DBS-based screening. This approach may prove effective in identifying cCMV in the context of screening programs, although it does depend upon the establishment of an infrastructure for collecting, processing, and following up on screen-positive urine samples [30]. A novel urine collection kit using filter paper demonstrated that this may be a promising approach for newborn screening for cCMV [31]. Future studies on newborn screening using both saliva [32,33] and urine collected on filter paper cards could hold promise in enhancing cCMV screening programs, and these merit additional study, but the established infrastructure for processing newborn DBS cards that exists in state newborn screening programs in the USA justifies adoption of universal cCMV using DBS PCR at the current time, challenges in sensitivity notwithstanding, as is now standard practice in Minnesota.

Our study stands out by specifically assessing the sensitivity of ddPCR in the context of DBS samples collected at birth, contributing to a better understanding of ddPCR’s performance in this specific clinical scenario. We have found that the sensitivity of ddPCR is lower than real-time PCR for CMV DNA detection in the DBS. For newborn screening, the priority is identifying infants with cCMV rather than obtaining a precise viral load measurement. Any amount of CMV DNA is considered abnormal and triggers further diagnostic testing to confirm cCMV infection. Researchers and clinicians need to consider the specific characteristics of the virus and the context of the analysis when selecting the appropriate PCR platform for their needs. Cost and throughput are also important when selecting a platform for CMV newborn screening. ddPCR is more expensive and time-consuming per sample than real-time PCR [15]. Further optimization of ddPCR methodology may be required to make this approach amenable to universal cCMV screening.

## 5. Conclusions

This study evaluated the sensitivity of ddPCR compared to quantitative real-time PCR for detecting CMV DNA in the newborn DBS. Singleplex UL55-HEX ddPCR exhibited the lowest limit of detection (best sensitivity) among the primer/probe combinations tested for ddPCR. However, when ddPCR was compared to real-time PCR in infants with confirmed cCMV, it demonstrated a lower level of clinical sensitivity. These results suggest that ddPCR does not demonstrate any enhancement in sensitivity for universal cCMV newborn screening programs compared to the use of real-time PCR testing of DNA eluted from DBS for use as a clinical screening tool. As universal screening programs move forward in clinical practice (for example, the recent initiation of universal cCMV screening in Minnesota in 2023 [7]), our findings highlight the importance of choosing optimal molecular diagnostic technologies for effective detection of cCMV in infants. Based on this study, we found no enhancement of sensitivity, and hence no clear advantage, for the use of ddPCR for cCMV DBS-based screening.

## Figures and Tables

**Table 1:** Primers/probes sets utilized for qPCR and ddPCR.

Gene	Primer/Probe	Sequence	Final Concentration	
			qPCR	ddPCR
UL83	Forward	GGA CAC AAC ACC GTA AAG C	0.4 μM	0.9 µM
	Reverse	GTC AGC GTT CGT GTT TCC CA	0.4 μM	0.9 µM
	Probe	FAM-CCC GCA ACC CGC AAC CCT TCA T-BHQ1		0.250 µM
	Probe	CAL610-CCC GCA ACC CGC AAC CCT TCA T-BHQ2	0.1 μM	
NRAS	Forward	GCC AAC AAG GAC AGT TGA TAC AAA	0.4 μM	
	Reverse	GGC TGA GGT TTC AAT GAA TGG AA	0.4 μM	
	Probe	FAM-ACA AGC CCA CGG AAC TGG CCA AGA-BHQ1	0.1 μM	
UL55	Forward	TGG GCG AGG ACA ACG AA		0.9 µM
	Reverse	TGA GGC TGG GAA GCT GAC AT		0.9 µM
	Probe	HEX-TGG GCA ACC ACC GCA CTG AGG-BHQ1		0.250 µM

**Table 2:** Comparison of limits of detection of single UL83 and UL55 and multiplex UL83 and UL83 ddPCR using CMV TowneΔ147 BAC DNA as a standard for dilutions.

Towne Dilution (Copies/PCR Reaction)	Real-Time UL83		ddPCR UL83 Singleplex		ddPCR UL55 Singleplex		ddPCR UL83 Multiplex		ddPCR UL55 Multiplex	
	Tests	Copies/Reaction	Tests	Copies/Reaction	Tests	Copies/Reaction	Tests	Copies/Reaction	Tests	Copies/Reaction
**2245.0**	20/20	2965.0	20/20	2252.1	20/20	2476.9	20/20	2150.3	20/20	2423.8
**1122.5**	20/20	1463.1	20/20	1121.8	20/20	1262.5	20/20	1065.8	20/20	1191.6
**561.3**	20/20	827.4	20/20	562.8	20/20	622.0	20/20	555.9	20/20	608.3
**280.6**	20/20	381.4	20/20	289.9	20/20	321.0	20/20	272.4	20/20	300.7
**140.3**	20/20	189.8	20/20	149.1	20/20	162.9	20/20	145.4	20/20	161.7
**70.2**	20/20	103.2	20/20	67.5	20/20	88.2	20/20	66.1	20/20	75.7
**35.1**	20/20	49.6	20/20	33.6	20/20	38.5	20/20	36.4	20/20	39.5
**17.5**	20/20	25.4	20/20	18.6	20/20	19.0	20/20	20.7	20/20	20.7
**8.8**	20/20	13.6	19/20	9.5	20/20	10.2	18/20	10.2	18/20	10.2
**4.4**	20/20	8.5	17/20	5.8	12/20	5.1	17/20	5.2	16/20	6.7
**2.2**	20/20	5.7	8/20	4.1	6/20	4.8	9/20	3.9	5/20	4.0
**1.1**	19/20	3.9	3/20	4.5	2/20	5.1	3/20	3.9	2/20	3.3
**0.5**	6/20	3.2	2/20	3.3	0/20	-	0/20	-	1/20	3.2
**0.3**	4/20	2.1	1/20	3.4	0/20	-	0/20	-	2/20	3.3

Tests: number of replicas for each assay (positives/tested). Copies/reaction: average copies per PCR reaction calculated by the qPCR or ddPCR for each dilution.

**Table 3:** Comparison of qPCR UL83, ddPCR multiplex UL83 and UL55, and ddPCR singleplex UL83 and UL55.

	Real-Time PCR	Multiplex ddPCR		Singleplex ddPCR	
Sample Number	UL83-Red610 (Copies/PCR Reaction)	UL83-FAM (Copies/PCR Reaction)	UL55-HEX (Copies/PCR Reaction)	UL83-FAM (Copies/PCR Reaction)	UL55-HEX (Copies/PCR Reaction)
**1**	ND	ND	ND	ND	ND
**2**	ND	ND	ND	ND	ND
**3**	ND	ND	ND	ND	ND
**4**	ND	ND	ND	ND	ND
**5**	**11.3**	ND	**3.5**	ND	ND
**6**	ND	ND	ND	ND	ND
**7**	ND	ND	ND	ND	ND
**8**	ND	ND	ND	ND	ND
**9**	ND	ND	ND	ND	ND
**10**	**1630**	**72.8**	**145.5**	**28.3**	**113.5**
**11**	**7.2**	ND	ND	ND	ND
**12**	ND	ND	ND	ND	ND
**13**	**35.5**	ND	ND	**3.1**	**5.2**
**14**	**1.5**	**4.8**	**6.4**	**3.1**	**9.9**
**15**	ND	ND	ND	ND	ND
**16**	**17.7**	**8.3**	**6.7**	**15**	**16.9**
**17**	ND	ND	ND	ND	ND
**18**	**1.5**	**6**	**14.9**	**11.4**	**13.4**
**19**	ND	ND	ND	ND	ND
**20**	**1.6**	**3.2**	**9.6**	**3**	**8.1**
**21**	ND	ND	ND	ND	ND
**22**	**1.4**	**4.5**	**26.8**	**3.4**	**12.9**
**23**	ND	ND	ND	ND	ND
**24**	ND	ND	ND	ND	ND
**25**	ND	ND	ND	ND	ND
**26**	ND	ND	ND	ND	ND
**27**	ND	ND	ND	ND	ND
**28**	ND	ND	ND	ND	ND
**29**	**1.5**	ND	ND	ND	ND

ND: not detected.

**Table 4:** Contingency table comparing qPCR UL83 and ddPCR UL55 in children with cCMV. A positive result in real-time PCR was confirmed when at least two out of three replicates exhibited a cross-point value of 40 or less. For ddPCR, a positive outcome was determined by the presence of two or more droplets above the threshold of detection.

Real-Time PCR UL83		**ddPCR UL55**		
		**Positive**	**Negative**	**Total**
	Positive	29	10	39
	Negative	0	10	10
	Total	29	20	49

## Data Availability

Data are available upon request from the corresponding author.
